# Therapeutic efficacy of dendritic cell vaccination in a novel syngeneic mouse model of diffuse hemispheric glioma, H3 G34-mutant

**DOI:** 10.1007/s11060-026-05545-z

**Published:** 2026-04-02

**Authors:** Geoffrey C. Owens, Erick M. Contreras, Jenny C. Kienzler, Janet Treger, Horacio Soto, Joey R. Orpilla, Chloe Qiao, Julia W. Chang, Alexander Lee, Wi-Jin Kim, Matthew Z. Sun, Sophie F. Peeters, Jacob A. Bethel, Aditya M. Kondajji, Eric C. Holland, Oren J. Becher, Linda M. Liau, Robert M. Prins, Anthony C. Wang

**Affiliations:** 1 Department of Neurosurgery, David Geffen School of Medicine at the University of California Los Angeles, Los Angeles, CA USA; 2 Division of Neuropathology, Department of Pathology and Laboratory Medicine, David Geffen School of Medicine at the University of California Los Angeles, Los Angeles, CA USA; 3 Department of Department of Molecular and Medical Pharmacology, David Geffen School of Medicine at the University of California Los Angeles, Los Angeles, CA USA; 4 Jonsson Comprehensive Cancer Center, University of California Los Angeles, Los Angeles, CA USA; 5 Parker Institute for Cancer Immunotherapy, Los Angeles, CA USA; 6 Fred Hutchinson Cancer Research Center, Seattle, WA USA; 7 Department of Pediatrics, Icahn School of Medicine at Mount Sinai, New York, NY USA; 8 Present Address: Institute of Experimental Immunology, University of Zurich, Zurich, Switzerland

**Keywords:** Diffuse hemispheric glioma, Dendritic cell vaccine, PD-1 checkpoint blockade, Syngeneic mouse tumor, Extracellular matrix

## Abstract

**Purpose:**

The prognosis for pediatric high-grade gliomas associated with mutations in the *H3-3A* gene is very poor. To investigate whether tumor lysate-pulsed dendritic cells (DC) together with checkpoint blockade might be a potential treatment modality for diffuse hemispheric glioma H3 G34-mutant (DHG), we have developed a novel syngeneic mouse model.

**Methods:**

We used the RCAS/tv-A system to target the expression of H3G34R and PDGFβ and knock out p53 in neural progenitors in C57BL/6 neonatal mice. Three independent cell lines were obtained that expressed transcripts associated with oligodendrocyte and interneuron lineages. Lethal tumor developed following intracranial injection.

**Results:**

Two cycles of DC vaccination with PD-1 blockade decreased tumor burden and increased survival. In treatment resistant tumors we found higher expression of several genes involved in remodeling the extracellular matrix compared with tumors from untreated animals, suggesting a causal link to resistance to immunotherapy in this tumor model.

**Conclusion:**

Immunotherapy involving autologous dendritic cells pulsed with tumor lysate and combined with anti-PD-1 antibody might be an effective treatment for DHG. Treatment failure in our tumor model is associated with increased expression of genes implicated in remodeling extracellular matrix in the tumor microenvironment.

**Supplementary Information:**

The online version contains supplementary material available at 10.1007/s11060-026-05545-z.

## Introduction

High-grade gliomas (HGG) are among the most lethal human cancers with median overall survival typically under two years from initial diagnosis [1, 2]. Recurrence after surgery is almost universal because tumor cells infiltrate the surrounding brain making a complete surgical excision essentially impossible. Pre-clinical studies involving checkpoint blockade and active vaccination have shown promise in addressing the problem of recurrence in HGG [3]. HGG seen in adult patients tend to be heterogeneous and often involve copy number variability, without highly conserved truncal mutations suitable for immune targeting [4]. HGG are generally low in somatic mutational burden [5], which is frequently a negative predictor of response to immunotherapy [6].

There are reasons to believe that the pediatric forms of HGG will respond differently to immunotherapy than adult forms of HGG, as they consistently carry a small number of oncogenic driver mutations [7]. Juvenile presentation of HGG has been shown to frequently involve somatic mutations in the *H3-3A* gene, which encodes a histone H3 variant [7, 8]. Missense mutations at codons K27 or G34 have divergent effects on gene expression [9, 10] that is context-dependent [11–13]. Diffuse hemispheric glioma, H3 G34-mutant (DHG) display distinct characteristics that differentiate it from the better-characterized diffuse midline glioma H3 K27-altered (DMG). DHG most commonly occurs later in childhood, and is typically cortical and lobar in location, in contrast with the midline or diencephalic location of DMG. *TP53* and *ATRX/DAXX* mutations have been reported in 95% and 84% of DHG respectively, with *PDGFRA* alterations in 44% of newly diagnosed tumors, and 81% at recurrence [14–17].

Cellular profiling of resected DHG has revealed that they are relatively devoid of infiltrating immune cells [18], and indicate that there may be few immunogenic tumor-specific antigens (TSAs), however recent work indicates that DHG may be amenable to immune therapy [19]. In order to study susceptibility of DHG to immunotherapy, we used the **R**eplication-**C**ompetent **A**vian leukosis virus long terminal repeat with **S**plice acceptor (RCAS)/tumor virus-a (tv-a) system [20, 21] to generate syngeneic H3G34R pediatric HGG lines. We then utilized this immunocompetent model to test the efficacy of targeted dendritic cell (DC) vaccination in combination with anti-PD-1 checkpoint blockade in pre-clinical experiments.

## Results

### Generation of syngeneic murine H3G34R pediatric high-grade gliomas

**Fig. 1:**
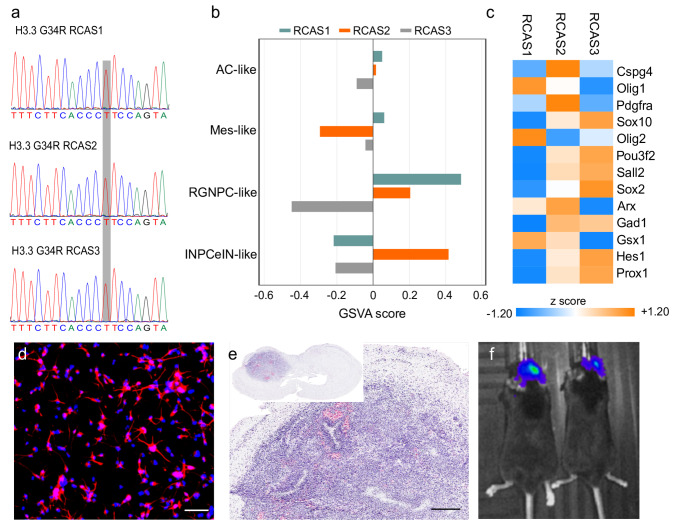
DHG model. (**a**) Chromatograms showing the presence of the G34R mutation in three independently derived RCAS/H3G34R tumor lines. The G to A transition results in a T on the opposite strand (highlighted in grey). The *H3-3A* G34R sequence was amplified with primers that flanked the transgene within the integrated RCAS provirus. (**b**) Bar graph showing Gene Set Variation Analysis (GSVA) scores derived by comparing the normalized filtered transcripts from the three RCAS cell lines with gene signatures derived from single cell RNA-sequencing human DHG G34R tumors. Ac-like, astrocyte-like cells; Mes-like, mesenchymal-like cels; RGNPC, radial glia-like to neuron progenitor-like cells; INPCeIN, interneuron progenitor to early GABAergic interneuron-like cells (**c**) Heat map of z scores calculated from the normalized transcript data showing variable expression between the RCAS/H3G34R lines of selected genes linked to oligodendrocyte progenitor cells (*Cspg4*,* Olig1*,* Pdgfra*,* Sox10*), neural stem cells (*Olig2*,* Pou3f2*,* Sall2*, and *Sox2*), and development of GABAergic interneurons (*Arx*,* Gad1*,* Gsx1*,* Hes1*, and *Prox1*). (**d**) Immunofluorescence image of RCAS2 cells stained for nestin. Scale bar = 50 microns (**e**) Hematoxylin/eosin-stained coronal section of cerebral cortex showing the development of a large tumor mass after implantation of RCAS2/H3G34R cells. Scale bar = 250 microns. The inset shows the position of the tumor in a coronal section of the whole brain. (**f**) Bioluminescence signal in the brains of mice implanted with RCAS2/H3G34R cells

Intraventricular injections of DF-1 cells producing viruses encoding *PDGF-B*, Cre recombinase, and *H3-3A G34R* into postnatal day 2–3 C56Bl/6J *Tp53*^fl/fl^ *Tg (Nestin-tv-A)* mice were made, and three non-clonal tumor lines were established from three different animals. The presence of the H3G34R mutation in each independently derived line was confirmed by genomic PCR using a primer set that would only amplify the inserted *H3-3 A G34R* transgene (Fig. 1a). We used Gene Set Variation Analysis (GSVA) to compare RNA transcripts from the three lines (Table S1) with gene signatures derived from single cell RNA-sequencing 14 human DHG G34R tumors [22]. On a scale of -1 to + 1 positive scores indicate higher expression of genes associated with a gene signature. RCAS1 and RCAS2 cells had attributes of radial glia neural progenitor-like cells; in addition, RCAS2 cells expressed genes associated with a metaprogram that defines an interneuron-like progenitor (Fig. 1b and Fig. S1). As shown in Fig. 1c, genes that define oligodendrocyte progenitor cells (*Cspg4*,* Olig1*,* Pdgfra*,* Sox10*) [23, 24], and stemness (*Olig2*,* Pou3f2*,* Sall2 and Sox2*) [25] were expressed in the three RCAS-derived H3G34R lines (RCAS/H3G34R) (Fig. 1c). Figure 1c also shows the expression of transcription factors involved in specifying GABAergic interneurons (*Arx*,* Gsx1*,* Hes1*,* and Prox1*) [26–28], and *Gad1*, the gene encoding a glutamate decarboxylase, which catalyzes the synthesize of gamma-aminobutyric acid from glutamate. All three lines expressed nestin RNA (Table S1), and protein expression in RCAS2 cells was confirmed by immunostaining (Fig. 1d). The RCAS2 cell line showed the highest concordance with both RGNPC and INPCeIN gene signatures and was selected for the in vivo experiments. Orthotopic delivery of RCAS2 cells expressing firefly luciferase resulted in the robust growth of intracranial tumors after 28 days (Fig. 1e and f).

### Treatment with tumor lysate-pulsed dendritic cells and anti-PD-1 monoclonal antibodies provides a survival benefit

**Fig. 2:**
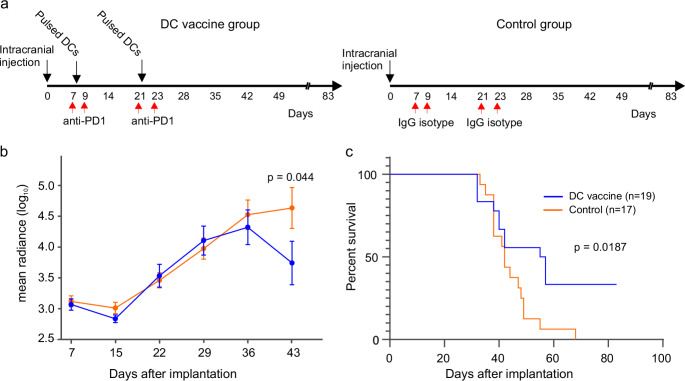
DC vaccination provides survival benefit (**a**) Schematic outline of the protocol used to treat animals after orthotopic placement of RCAS/H3G34R tumor cells. (**b**) Tumor growth monitored by in vivo imaging (mean radiance within region of interest in photons/second/cm^2^/solid angle). (**c**) Kaplan-Meier plot showing a significant survival benefit over time after DC vaccination combined with PD-1 blockade (*p* = 0.0187, Mantel-Cox test)

We next asked whether DC vaccination together with checkpoint blockade would provide a survival benefit in our syngeneic DHG model. Forty animals were implanted with RCAS/H3G34R tumor cells via intracranial injection and randomized into two groups. After 7 days the presence of tumors was assessed by luciferase-catalyzed bioluminescence. Animals with evidence of tumor (*n* = 19) were injected intradermally with 10^6^ bone marrow-derived DCs pulsed with tumor lysate, followed by an intraperitoneal injection of PD-1 monoclonal antibody (mAb). Control tumor-bearing animals (*n* = 17) received an IgG1 isotype mAb without DC vaccination. PD-1 mAb and isotype mAb were administered again 48 h later [29]. The treatment group received a second round of DC vaccination and PD-1 mAb after 14 days (Fig. 2a). Tumor growth was monitored by measuring bioluminescence, and we observed a decrease in average tumor size between the treated and control 22 days after the second DC vaccination, coincident with the loss of more control animals (Fig. 2b), after which we stopped the in vivo imaging. As shown in Fig. 2c DC vaccinated animals survived longer than any of the animals in the control group. All the surviving mice were sacrificed on day 83.

### Increased number of CD8^+^ T cells in tumors treated with tumor lysate-pulsed dendritic cells

**Fig. 3:**
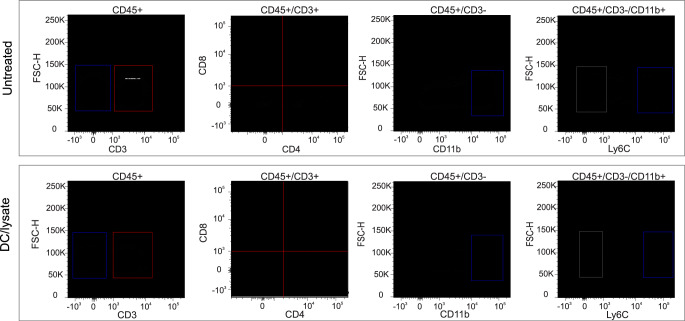
Increased CD8^+^ T cells in TIL fractions from DC-treated animals. Seven days after the second DC vaccination, TILs were isolated from the brains of treated and untreated animals (two animals per group) and analyzed by flow cytometry. TILs were stained with a panel comprising CD45, CD3, CD4, CD8, CD11b, and Ly6C antibodies

We have previously shown that the survival benefit of DC vaccination with concurrent PD-1 blockade is completely dependent upon CD8^+^ T cells [29]. To determine whether DC vaccination plus PD-1 inhibition resulted in increased T cells in implanted RCAS/H3G34R tumors we repeated the first in vivo experiment and sacrificed tumor-bearing animals on day 7 after the second injection of DCs (28 days after tumor implantation). Brains were dissociated to obtain an immune cell-enriched fraction. As shown in Fig. 3, the proportion of CD8^+^ T cells in CD45^+^/CD3^+^ cells in tumor infiltrating leukocytes (TILs) from animals treated with DCs pulsed with tumor lysate was markedly higher. CD3^−^/CD11b^+^ myeloid cells were predominantly Ly6C^+^, indicating that they were likely derived from circulating monocytes (Fig. 3).

### Increased expression of genes associated with remodeling the extracellular matrix in animals that failed treatment

Although the immunotherapy protocol appeared to be effective in our tumor model, only a quarter of the treated mice survived to the end of the experiment. To investigate the cause of treatment failure, we dissected the tumors from three treated animals that had succumbed to the tumor burden after the second DC vaccine, and tumors from three untreated animals that died in the same timeframe (40 to 60 days post implantation) and extracted RNA for RNAseq. The estimated tumor purity of all six samples was approximately the same (58% ± 2.5%) [30].

**Fig. 4:**
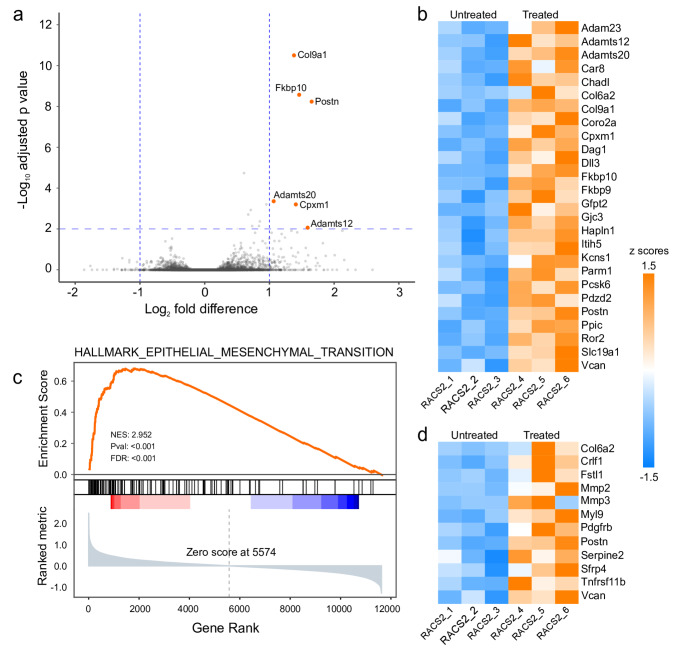
RNA transcripts associated with treatment failure encode extracellular matrix genes. (**a**) Volcano plot showing the results of the pairwise comparison between RNA transcripts between tumors taken from treated and untreated animals that succumbed to tumor burden. The top 6 genes overrepresented in the treated tumors are shown in orange. (**b**) Heat map of the z scores calculated from the normalized counts of 27 genes whose expression was significantly higher in treatment resistant tumors compared to untreated tumors (padj < 0.05; 1.5-fold difference). (**c**) GSEA plot showing significant enrichment in an epithelial to mesenchymal transition gene signature in treatment resistant tumors. Nominal p-value and false discovery rate are calculated from a permutation statistic (**d**) Heat map of the z scores calculated from the normalized counts of the highest ranked genes (metric score > 1). Vcan, Postn, and Col6a2 are found in both heatmaps

Principal Component Analysis (PCA) using all normalized transcript counts did not show any obvious similarity between each sample (Fig. S2). However, by grouping the treated and untreated samples and applying differential gene expression analysis (DESeq2, adjusted p-value of < 0.01 and > 2-fold difference) we identified six genes, *Col9a1*, *Fkbp10*, *Postn*,* Adamts12*, *Adamts20* and *Cpxm1* with higher levels of expression in the tumors taken from treated tumor-bearing mice compared with tumors from untreated animals (Fig. 4a). *Col19a1* encodes an alpha chain of Type IX collagen a fibril-associated collagen with interrupted triple helices (FACIT) collagen [31]; *Fkb10* encodes FK506 binding protein 65, a peptidyl-prolyl cis-trans isomerase that catalyzes a rate limiting step in triple helical procollagen formation [32]; *Postn* encodes periostin, a secreted signaling protein found in extracellular matrix (ECM) [33]; ADAMTS12 and 20 are extracellular metalloproteases [34], and CPXM1 is a secreted collagen-binding protein [35]. Higher levels of POSTN, FKBP10, CPXM1, and COL9A1 mRNAs in brain tumors are associated with shorter survival times [36] (Fig. S3).

By relaxing the significance and fold difference thresholds to *p* < 0.05 and 1.5-fold difference, we identified an additional 21 genes (Fig. 4b). PCA using these genes clearly partitioned the treated and untreated tumor samples (Fig. S2). Postn was the most connected node in a network constructed from Pearson correlation coefficients > 0.9, between these differentially expressed genes (Fig. S4a). Gene set enrichment analysis [37] showed a significant enrichment in a gene signature associated with epithelial to mesenchymal transition (EMT) (Fig. 4c), and periostin was among the top ranked genes in this gene signature (Fig. 4d). Immunocytochemistry confirmed the expression of periostin in implanted RCAS/H3G34 tumors (Fig. S4b).

## Discussion

In this report, we provide preclinical support for treating DHG with tumor-targeted DCs. In our survival experiment, combined tumor lysate-pulsed DCs and PD1 blockade provided a demonstrable therapeutic benefit. This personalized treatment approach does not require the identification of specific neoantigens and has been shown to result in extended survival of some adult patients with recurrent HGG [38]. Although it has been reported that oncohistone-driven pediatric HGG in the HERBY Phase II trial were immunologically cold [39], a peptide that spans the H3K27M mutation has been shown to elicit a CD8^+^ T cell response in patients with DMG [40]. While the HLA-A*02 restricted peptide vaccine alone may be not therapeutically effective [41], a longer K27M-spanning peptide targeting a CD4^+^ T cell response showed significant promise [42]. The H3G34R/V mutant protein has not been shown to serve as an immunogenic tumor specific antigen; however, our experiments demonstrate that DHG might be, in principle, susceptible to this type of immunotherapy.

In the syngeneic murine tumor model of DHG that we developed autocrine PDGF signaling was driven by introducing a transgene encoding PDGFβ, which has been shown to lead to the development of high grade gliomas in the context of inactivating or suppressing CDKN2A or P53 [24, 43]. It has been reported that while H3G34R may be necessary to transform neural progenitor cells, it may not be required for persistence of the transformed state [13], which may involve activating *PDGFRA* mutations or *PDGFRA* copy number amplification [17]. Further work is required to determine the relative contributions of the *H3G34R* transgene and PDGF signaling to oncogenesis in our tumor model.

Recent reports provide evidence that the cell of origin of DHG G34 tumors is a ventral forebrain interneuron progenitor [17, 22]. The RCAS/H3G34R lines we generated expressed transcripts linked to both interneuron and oligodendroglia lineages, and to transcripts encoding TFs that maintain neural stem cells in a proliferative and uncommitted state [25]. Both neuronal and glia progenitors are present in the subventricular zone (SVZ) of early postnatal mouse brain [44], and would be the likely targets of the viruses produced by the DF-1 cells that we injected. In future work employing single cell RNA-sequencing we will determine whether genes linked to each lineage are expressed by the same cells. It should be noted that the different gene signatures derived from single cell RNA sequencing DHG GR34 tumors that we used in GSVA attests to the heterogeneity of these tumors [22].

Starting from an approximately equal tumor burden, about a quarter of the animals that were treated survived to the stopping point of the experiment while the rest did not. This difference might be attributable to technical variability; however, we obtained transcriptomic data from non-survivors to indicate that changes in gene expression may account for therapeutic failure. Specifically, we found that genes associated with ECM were significantly higher in treated non survivors compared with untreated non survivors suggesting the possibility of an active response by the tumor to the immunotherapy that involves remodeling the ECM. Periostin is the top ranked gene in the EMF gene signature and Postn transcripts were significantly higher in the treatment resistant tumors. Periostin promotes procollagen maturation via interaction with BMP-1 (Bone morphogenetic protein-1, pro-collagen C-proteinase), which activates lysyl oxidase preprotein (LOX) by cleaving the N-terminal propeptide [45]. LOX catalyzes the covalent crosslinking of collagen fibrils, thus periostin promotes the assembly of triple helical collagen [45]. It has been reported that periostin secreted by glioma stem cells recruits immunosuppressive macrophages to the TME via integrin binding [46], and we detected Postn RNA transcripts in our RCAS/HG34R cell lines (Table S1). Stromal cells are also a source of periostin [47]. Higher POSTN mRNA in brain tumors is associated with poorer patient survival (Fig. S3), and resistance to temozolamide by glioblastomas is correlated with increased expression of periostin [48]. Pre-clinical studies with peripheral tumor models have shown that specifically blocking periostin with an antibody [49, 50] or a DNA aptamer [51] inhibited tumor growth and metastasis.

## Materials and methods

### Derivation of a model of diffuse hemispheric glioma, H3 G34-mutant

All animal experimental protocols were approved by the UCLA Institutional Animal Care and Use Committee. Surgical procedures were performed in a BSL2 biological safety cabinet. RCAS/H3G34R were generated similarly to Misuraca et al. [21]. Postnatal day 2–3 pups were anesthetized with a mixture isofluorane/oxygen delivered through a nose cone trimmed to fit the neonate. Two microliters of packaging cells comprising equal numbers of DF1 cells producing RCAS viruses encoding H3G34R, Cre recombinase, and PDGF-B in phosphate-buffered saline (PBS) were injected (1.0 × 10^5^ cells per µl) into the left lateral ventricle through a 28-gauge needle attached to a 10 µl Hamilton syringe. The needle penetrated the skull to a depth of 2 mm at a location 0.25 mm lateral to the sagittal suture and 0.5–0.75 mm rostral to the neonatal coronary suture. Animals were returned to their home cages and monitored daily after weaning for signs of tumor formation as evidenced by loss of body weight and problems with locomotion, at which point the animal was euthanized with CO_2_, and the brain removed. The Brain Tumor Dissociation Kit (P) (Miltenyi Biotec Inc., Auburn, CA) was used to dissociate mouse brains, which were then fractionated on a 70%/30% Percoll^®^ gradient (Sigma-Aldrich, St. Louis, MO) to the remove myelin. Cells were placed in culture in medium formulated to support the growth of human pediatric DMG [52]: 1: 1 DME/F12: Neural Basal medium, 1x Minimum Essential Amino Acids, sodium pyruvate (10mM), HEPES (10mM), 1x GlutaMAX and EGF (10 ng/ml), bFGF (10 ng/ml), PDGFa/b (10 ng/ml) (ThermoFisher, Waltham, MA), and heparin solution (5 IU/ml; Sigma Aldrich St. Lous, MO). All injected animals were sacrificed after eighteen weeks.

### Orthotopic delivery of tumor cells and treatment protocol

RCAS/H3G34R tumor cells were expanded and transduced with a third generation VSV-G pseudo-typed HIV-based recombinant lentivirus encoding strawberry fluorescent protein and firefly luciferase open reading frames linked by an internal ribosome entry site, and under the transcriptional control of the CMV IE promoter. Under ketamine/xylazine anesthesia, a burr hole was drilled in the skull 2 mm lateral to Bregma and 0.5 mm caudal and injections of 1 × 10^5^ RCAS/H3G34R tumor cells in 2 µL of PBS were made into the left hemispheres of eight-week-old syngeneic C57Bl/6J female mice. Cells were injected through a 28-gauge needle attached to a 10 µl Hamilton syringe at a depth of 2.5 mm. DCs were prepared from bone marrow (BM) essentially as previously described [29]. In brief, BM cells were plated in RPMI-1640 plus 10% fetal bovine serum (PBS) with antibiotics, and after 24 h the nonadherent cells were counted and re-plated in 12-well tissue culture plates (~ 2 × 10^6^ cells per well) in RPMI-1640 plus 10% FBS supplemented with murine granulocyte-macrophage colony stimulating factor (100 ng/ml; Biolegend, San Diego, CA) and interleukin-4 (500 U/ml; Biolegend). The medium was replaced after three days, and three days later the adherent differentiated DCs were pulsed overnight with 250 µg per well of a freeze thawed lysate of untransduced RCAS/H3G34R tumors cells per well. An aliquot of cells was stained with anti-mouse CD11c, F4/80, CD86 and I-A^d^ antibodies and analyzed by flow cytometry to confirm that they were differentiated DCs. Cells were harvested, washed, resuspended in PBS and 1 × 10^6^ cells were injected intradermally seven days after tumor implantation together with an IP injection of anti-mouse PD-1 (250 µg per animal; clone RMP1-14; Bio X Cell, Lebanon, NH). PD-1 mAb was re-administered 48 h later. The same treatment regimen was repeated 21 days after tumor implantation. In vivo imaging was performed under isoflurane anesthesia after IP injection of luciferin (100 µl of 5 mg/ml stock solution). Chemiluminescence images were captured using an IVIS Lumina II imaging system (Perkin Elmer, Waltham, MA) to measure tumor burden. Log transformed mean radiance values and standard errors, and survival data were analyzed and plotted in GraphPad Prism (GraphPad Software Corp., San Diego, CA). Graphs were exported as enhanced metafiles to CorelDraw2023 (Corel Corporation, Ottawa, Canada).

### DNA sequencing

Genomic DNA was isolated from cultured RCAS/H3G34R tumor cells using a commercially available kit (Quick-DNA miniprep kit, Zymo Research, Irvine CA). To confirm the presence of the G34R mutation, PCR primers flanking the cloning site in the RCAS vector were used to amplify inserted sequences (forward primer 5’ GTCTGTGTGCTGCAGGAGCTGAGCTGACTCTGCTG 3’, reverse primer 5’ GATACGCGTATATCTGGCCCGTACATCGCATCG 3’) using Q5^®^ DNA polymerase (New England Biolabs, Ipswich, MA) with the following conditions: 98 °C 2 min → 35 cycles (98 °C 15 s, 68 °C 30 s, 72 °C 30 s) → 72º C 2 min. PCR products were treated with ExoSAP-IT™ (ThermoFisher)) then sequenced using an H3.3-specific primer (5’GCACGTTCTCCACGTATGCGGCGTG 3’).

### RNA sequencing

Total RNA was extracted using a commercially available kit (Direct-zol RNA kit; Zymo Research) from mouse RCAS/H3G34R cells and RCAS/H3G34R tumors dissected from mouse brains. Libraries were constructed with a KAPA Stranded mRNA-Seq Kit (Roche Diagnostics Corp, Indianapolis, IN), and sequencing was performed on a HiSeq3000 sequencer to produce 50 base-pair single-end reads (1 × 50 bp) (Illumina Inc., San Diego, CA). Reads were aligned to genes in the mouse genome assembly GRCm38 (mm10) using STAR [53]. The R package DEBrowser was used to filter (< 1 counts per million) and normalize (Trimmed median of the M-values) [54] raw counts, and for differential gene expression using the filtered counts (DESeq2) [55].The R package GSVA was used for gene set variation analysis [56]; Graphia [57], Gene Set Enrichment Analysis [37, 58], and Morpheus by Broad Institute (RRID: SCR_017386) were also used. Graphical data were exported in pdf format to CorelDraw2023.

### Flow Cytometry

A leukocyte fraction was isolated from tumor-bearing animals by dissociating brain tissue as described above and fractionating on a 30%:70% Percoll^®^ gradient. The following mAbs were used to label cells: CD45 (Alexa Fluor (AF) 700-conjugated; clone 30-F11), CD3 (Allophycocyanin (APC)-Cyanine 7 (Cy7)-conjugated; clone 145-2C11), CD4 (APC-conjugated; clone RM4-5), CD8 (Phycoerythrin (PE)-Cy7-conjugated; clone 53 − 6.7), CD11b (PE-conjugated; clone M1/70, Ly6C (BV650-conjugated; clone Hk1.4), plus Zombie Violet™. DCs were stained both with CD11c (PerCP-Cyanine5.5-conjugated; clone) and F4/80 (FITC-conjugated, clone BM8), and with CD86 (PerCP-Cyanine5.5-conjugated; clone GL-1) and IA^d^ (FITC-conjugated; clone AF6-120.1). All antibodies were purchased from Biolegend (San Diego, CA), and samples were run on a LSRII analytical flow cytometer (Becton Dickinson, Franklin Lakes, NJ). Individual FCS files were analyzed using FlowJo^®^ software (FlowJo LLC, Ashland, OR); 2-D contour plots were exported as scalable graphics vector files to CorelDraw2023.

### Immunocytochemistry

Brains from tumor-bearing animals were fixed by immersion in formalin-free zinc fixative (Becton Dickinson, San Diego, CA), embedded in paraffin and sectioned. Deparaffinized sections were treated with hydrogen peroxide (3%) for 10 min., followed by antigen retrieval in 10 mM citrate buffer (pH 6.0 containing 0.05% Tween20) for 15 min. at 114–121 ℃ in a TintoRetriever Pressure Cooker (BioSB, Inc., Santa Barbara, CA). Sections were blocked and stained with a periostin mouse monoclonal antibody Ab (1:2000, ProteinTech Group, Inc., Rosemont, IL). Staining was visualized with an HRP-conjugated horse anti-mouse secondary Ab and 3, 3’-diaminobenzidine (ImmPRESS kit, Vector Laboratories, Inc., Newark, CA), Vector Laboratories, Inc., Newark, CA), followed by counterstaining with hematoxylin. Images were acquired using an Aperio ScanScope XT scanner (Aperio, Vista CA), then transferred to CorelPhoto-Paint, and rescaled from 72 dpi to 600 dpi. Brightness and contrast were adjusted in CorelDraw. RCAS2 were grown in a chamber slide on a poly L-lysine/laminin substratum (Life Technologies), then fixed with paraformaldehyde (4% in PBS), and blocked with 10% goat serum in Tris-buffered saline containing 0.3% Triton X100. Cells were incubated overnight at 4 °C with an anti-mouse nestin antibody (1:500, 10C2, Life Technologies) in blocking solution followed by washing then incubation in Alexa Fluor^®^ 568 goat anti-mouse secondary antibody (1:1000, Life Technologies) in blocking solution for 1 h at room temperature, and cover-slipping with ProLong^®^ Gold anti-fade reagent containing DAPI (Life Technologies). Fluorescent images were acquired with an Olympus spinning disk confocal microscope (Olympus America, Inc., Center Valley, PA) controlled by SlideBook™ image acquisition and analysis software (Intelligent Imaging Innovations, Inc. Denver CO). Images were transferred to CorelPhoto-Paint, and rescaled from 72 dpi to 600 dpi, and brightness and contrast were adjusted in CorelDraw.

## Supplementary Information

Below is the link to the electronic supplementary material.

Supplementary Material 1

Supplementary Material 2

Supplementary Material 3

Supplementary Material 4

Supplementary Material 5

Supplementary Material 6

## Data Availability

All data supporting the findings of this study are available within the paper and its Supplementary Information.
