# Supplementary material for: Therapeutic efficacy of dendritic cell vaccination in a novel syngeneic mouse model of diffuse hemispheric glioma, H3 G34-mutant
Source: J Neurooncol. 2026 Apr 2;177(2):88. doi: 10.1007/s11060-026-05545-z (PMC13046641; doi:10.1007/s11060-026-05545-z)
Supplement: Supplementary file 1 — Supplementary Material 1 [file 11060_2026_5545_MOESM1_ESM.pdf]

Therapeutic Efficacy of a Dendritic Cell Vaccine in a Novel Syngeneic Mouse Model of Diffuse Hemispheric Glioma, H3 G34-Mutant

Owens et al. Journal of Neuro-Oncology

Corresponding author: Anthony Wang, Dept. of Neurosurgery, David Geffen School of Medicine, UCLA
Email: acwang@mednet.ucla.edu

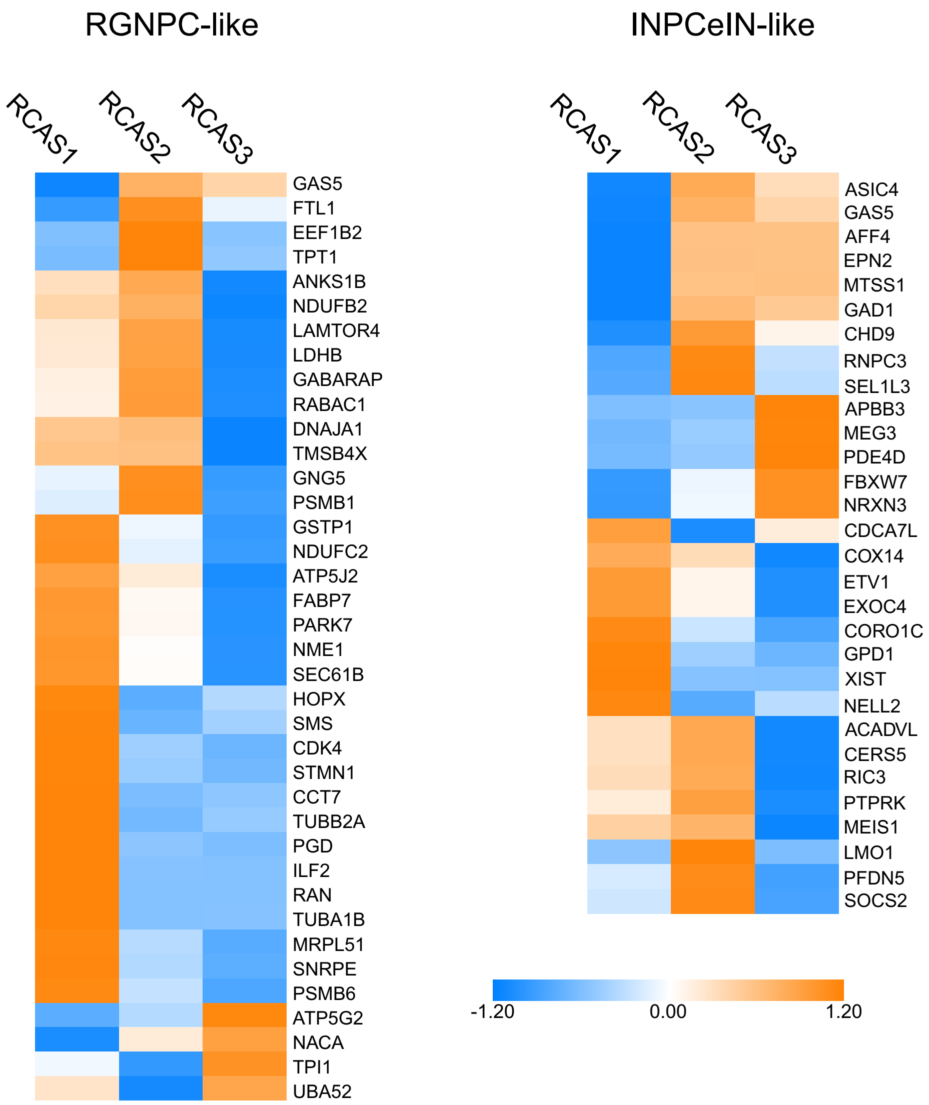

Fig. S1. Heatmaps showing the expression of genes in the three RCAS/H3G34R cell lines from the metaprogram gene signatures derived by Liu et al.[22] from frozen DHG-H3G34 tumor cell populations. In the case of the RGNPC-like metaprogram, 38/50 genes were found in the filtered and normalized RNAseq data (Table S1), and for the INPCeIN-like metaprogram, 30/50 genes were found. Genes were clustered hiearchically using one minus pearson correlation coefficients. Z scores were converted to colors on a scale from -1.2 to +1.2.
