# Supplementary material for: Therapeutic efficacy of dendritic cell vaccination in a novel syngeneic mouse model of diffuse hemispheric glioma, H3 G34-mutant
Source: J Neurooncol. 2026 Apr 2;177(2):88. doi: 10.1007/s11060-026-05545-z (PMC13046641; doi:10.1007/s11060-026-05545-z)
Supplement: Supplementary file 2 — Supplementary Material 2 [file 11060_2026_5545_MOESM2_ESM.pdf]

Therapeutic Efficacy of a Dendritic Cell Vaccine in a Novel Syngeneic Mouse Model of Diffuse Hemispheric Glioma, H3 G34-Mutant

Owens et al. Journal of Neuro-Oncology

Corresponding author: Anthony Wang, Dept. of Neurosurgery, David Geffen School of Medicine, UCLA
Email: acwang@mednet.ucla.edu

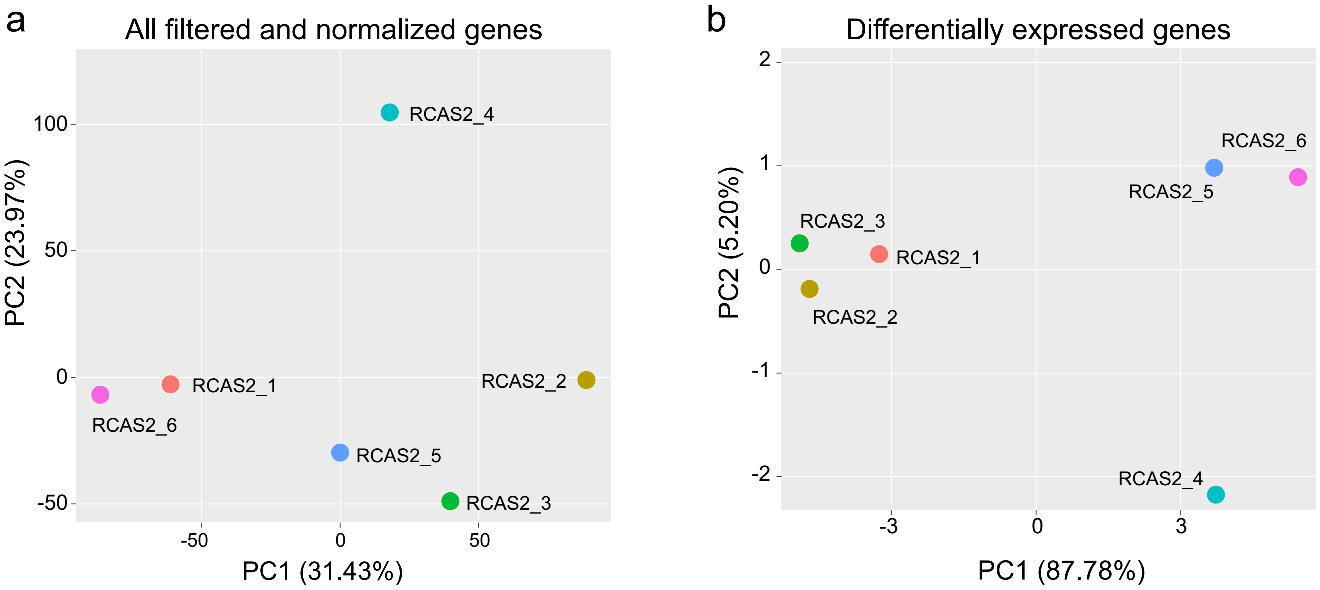

Fig S4. (a) PCA plot generated from the normalized expression values of all low count filtered genes from the untreated (RCAS2_1-3) and treated (RCAS2_4-6) groups. (b) PCA plot generated from the normalized expression values of the differentially expressed genes between the two groups (DESeq2 padj <0.05, 1.5-fold difference).
