# Supplementary material for: Therapeutic efficacy of dendritic cell vaccination in a novel syngeneic mouse model of diffuse hemispheric glioma, H3 G34-mutant
Source: J Neurooncol. 2026 Apr 2;177(2):88. doi: 10.1007/s11060-026-05545-z (PMC13046641; doi:10.1007/s11060-026-05545-z)
Supplement: Supplementary file 3 — Supplementary Material 3 [file 11060_2026_5545_MOESM3_ESM.pdf]

Therapeutic Efficacy of a Dendritic Cell Vaccine in a Novel Syngeneic Mouse Model of Diffuse Hemispheric Glioma, H3 G34-Mutant

Owens et al. Journal of Neuro-Oncology

Corresponding author: Anthony Wang, Dept. of Neurosurgery, David Geffen School of Medicine, UCLA
Email: acwang@mednet.ucla.edu

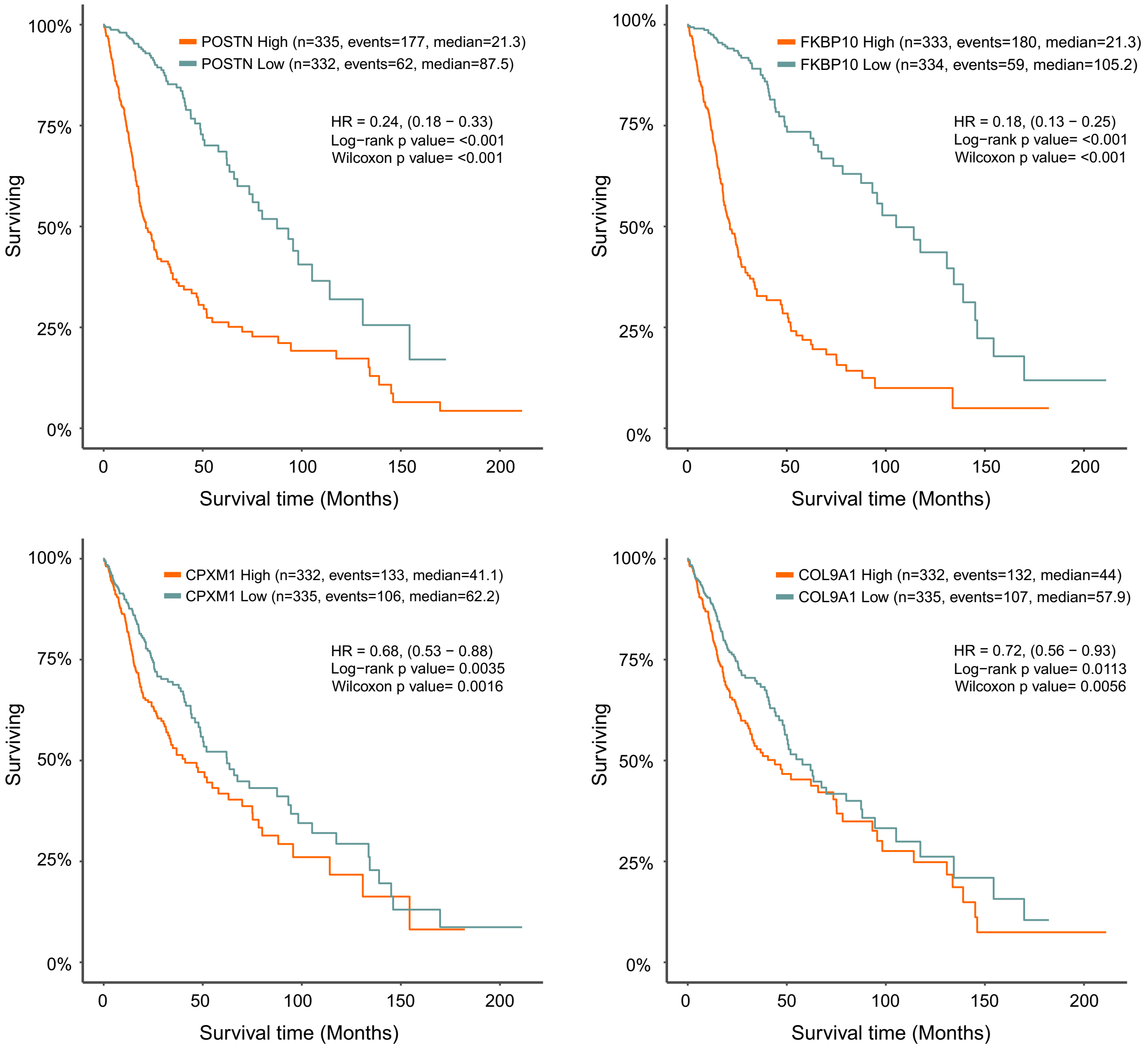

Fig. S3. Reduced survival of patients with brain tumors expressing higher levels of POSTN, FKBP10, CXPM1, and COL9A1 mRNAs. Survival curves for all histological types of brain tumor were generated using the GlioVis visualization toolkit.
