# Supplementary material for: Therapeutic efficacy of dendritic cell vaccination in a novel syngeneic mouse model of diffuse hemispheric glioma, H3 G34-mutant
Source: J Neurooncol. 2026 Apr 2;177(2):88. doi: 10.1007/s11060-026-05545-z (PMC13046641; doi:10.1007/s11060-026-05545-z)
Supplement: Supplementary file 4 — Supplementary Material 4 [file 11060_2026_5545_MOESM4_ESM.pdf]

Therapeutic Efficacy of a Dendritic Cell Vaccine in a Novel Syngeneic Mouse Model of Diffuse Hemispheric Glioma, H3 G34-Mutant

Owens et al. Journal of Neuro-Oncology

Corresponding author: Anthony Wang, Dept. of Neurosurgery, David Geffen School of Medicine, UCLA
Email: acwang@mednet.ucla.edu

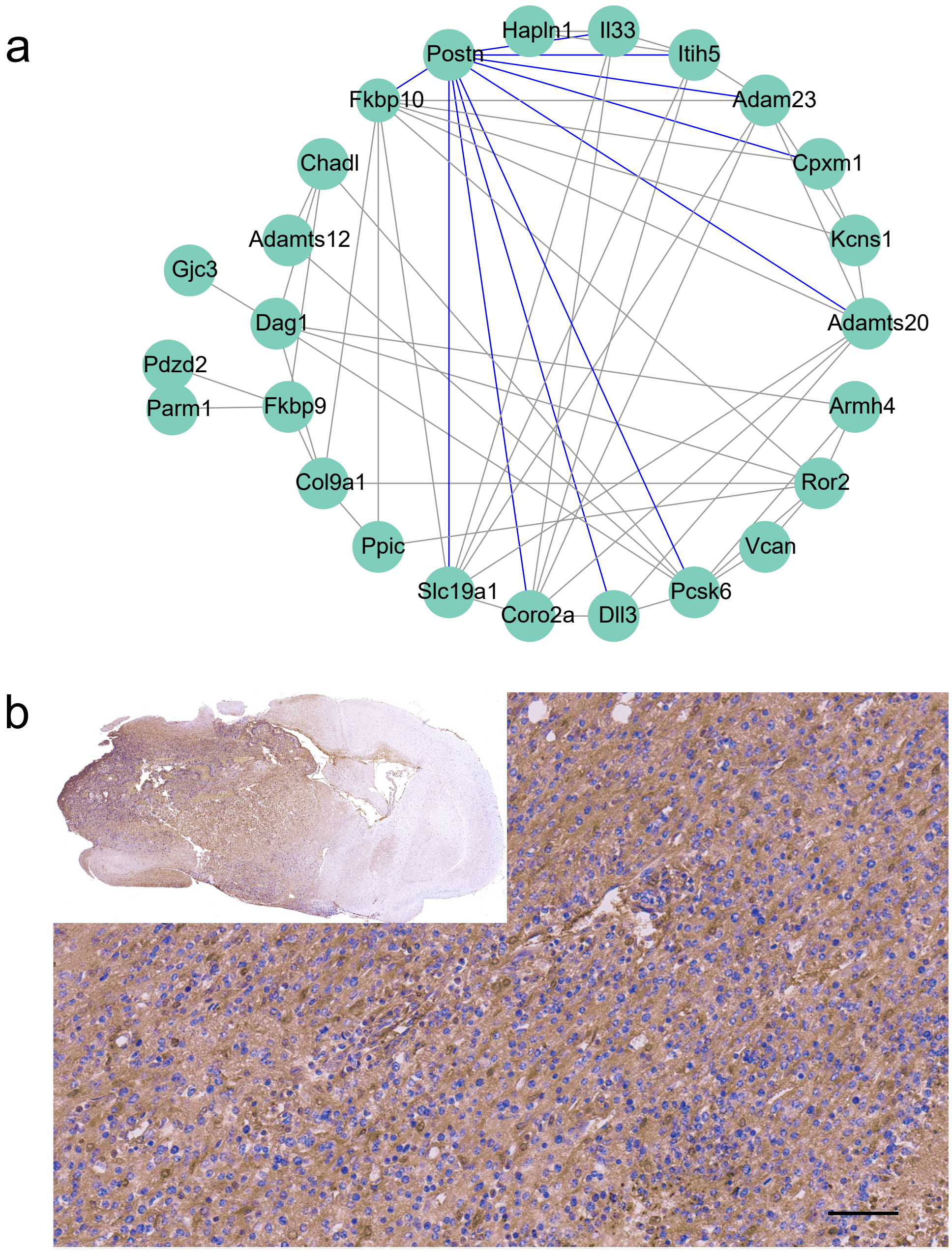

Fig. S4. (a) Graphical representation of the co-expression of the differentially expressed genes between treatment resistant and untreated tumors (DESeq2 padj<0.05 >1.5-fold difference). The graph was constructed from the normalized transcript counts of each gene and shows that Postn is the most connected gene. Edges correspond to Pearson correlation coefficients >0.9. (b) Coronal section showing periostin immunostaining in a large tumor mass of RCAS/H3G34R cells that occupies the left side of the brain (inset). The section was counterstained with hematoxylin. Scale bar = 50 microns.
